# ApoE4 expression accelerates hippocampus-dependent cognitive deficits by enhancing Aβ impairment of insulin signaling in an Alzheimer’s disease mouse model

**DOI:** 10.1038/srep26119

**Published:** 2016-05-18

**Authors:** Elizabeth S. Chan, Mahesh Shivarama Shetty, Sreedharan Sajikumar, Christopher Chen, Tuck Wah Soong, Boon-Seng Wong

**Affiliations:** 1 Departments of Physiology, Yong Loo Lin School of Medicine, National University of Singapore 117456, Singapore; 2 Memory Networks Program, Neurobiology and Ageing Program, Life Sciences Institute, National University of Singapore 117456, Singapore; 3 Pharmacology, Yong Loo Lin School of Medicine, National University of Singapore 117597, Singapore; 4 Memory Ageing and Cognition Centre (MACC), National University Health System (NUHS) 117599, Singapore

## Abstract

The apolipoprotein E4 (ApoE4) is the strongest genetic risk factor for Alzheimer’s disease (AD). The AD brain was shown to be insulin resistant at end stage, but the interplay between insulin signaling, ApoE4 and Aβ across time, and their involvement in memory decline is unclear. To investigate insulin response in the ageing mouse hippocampus, we crossed the human ApoE-targeted replacement mice with the mutant human amyloid precursor protein (APP) mice (ApoExAPP). While hippocampal Aβ levels were comparable between ApoE3xAPP and ApoE4xAPP mice at 26 weeks, insulin response was impaired in the ApoE4xAPP hippocampus. Insulin treatment was only able to stimulate insulin signaling and increased AMPA-GluR1 phosphorylation in forskolin pre-treated hippocampal slices from ApoE3xAPP mice. In ApoE4xAPP mice, insulin dysfunction was also associated with poorer spatial memory performance. Using dissociated hippocampal neuron *in vitro*, we showed that insulin response in ApoE3 and ApoE4 neurons increased AMPA receptor-mediated miniature excitatory postsynaptic current (mEPSC) amplitudes and GluR1-subunit insertion. Pre-treatment of ApoE3 neurons with Aβ42 did not affect insulin-mediated GluR1 subunit insertion. However, impaired insulin sensitivity observed only in the presence of ApoE4 and Aβ42, attenuated GluR1-subunit insertion. Taken together, our results suggest that ApoE4 enhances Aβ inhibition of insulin-stimulated AMPA receptor function, which accelerates memory impairment in ApoE4xAPP mice.

Alzheimer’s disease (AD) is a neurodegenerative disorder characterized by the gradual deterioration of memory and cognitive faculties, ultimately leading to dementia^1^. Pathologically, it is characterized by the accumulation of extracellular amyloid plaques and intracellular neurofibrillary tangles of hyperphosphorylated tau. While AD has multiple causes, inheriting the human apolipoprotein E4 (ApoE4) allele remains the strongest genetic risk factor^2^,^3^.

ApoE exists in three isoforms, namely ApoE2, ApoE3 and ApoE4, and differ from each other by having either a cysteine or arginine residue at position 112 and 158^4^. The ApoE3 genotype occurs in ~80% of the population; whilst ApoE4 and ApoE2 occurs in ~14% and <7% respectively in most populations. However, ApoE4 occurs in >50% of sporadic AD patients where its inheritance results in earlier onset of the disease^2^,^3^. Over the last two decades, several groups have reported the association of the apoE gene to memory and cognition^2^,^5^. ApoE4 expression was reported to impair synaptic plasticity and reduce neuronal surface expression of Apoer2, which binds to α-amino-3-hydroxy-5-methyl-4-isoxazolepropionic acid (AMPA) and *N*-methyl-D-aspartate (NMDA) receptors^6^. Moreover, we have reported that ApoE4 expression can reduce the phosphorylation of NMDA and AMPA receptors^7^, possibly impairing LTP in the ageing mice^8^. In AD, the expression of ApoE4 is linked to earlier onset of memory decline^9^,^10^. At present, its mode of action is still unclear.

Memory impairment in AD is gradual and insidious, making the onset of the disease difficult to discern. Interestingly, aberrant insulin signaling was identified as an early risk factor for the disease^11^, and the human AD brain was demonstrated to be at an insulin resistant state^12^,^13^. Furthermore, insulin response was shown to be crucial for learning and memory^14^. In mice models, functional insulin signaling reversed Aβ42 induced LTP impairment^15^,^16^. In clinical studies, increasing insulin administration improved the overall performance of AD individuals in learning and memory tasks. However, when the study cohort was stratified into their ApoE groups, ApoE4 carriers did not benefit from insulin treatment^14^,^17^. Taken together, it seems that there is an interplay between ApoE isoforms, insulin signaling and Aβ42, which could be involved in the underlying mechanism of memory decline in AD.

To investigate this, we crossed the human ApoE-targeted replacement mice^18^,^19^ with mice carrying human amyloid precursor protein (APP), bearing the Swedish (K670N/M671L) and the Indiana (V717F) mutations^20^, and examined insulin response in the hippocampus of the ageing ApoExAPP mice.

## Results

### Aβ deposition in the hippocampus of aged ApoE4xAPP was significantly higher than ApoE3xAPP and APP mice

**Figure 1:**
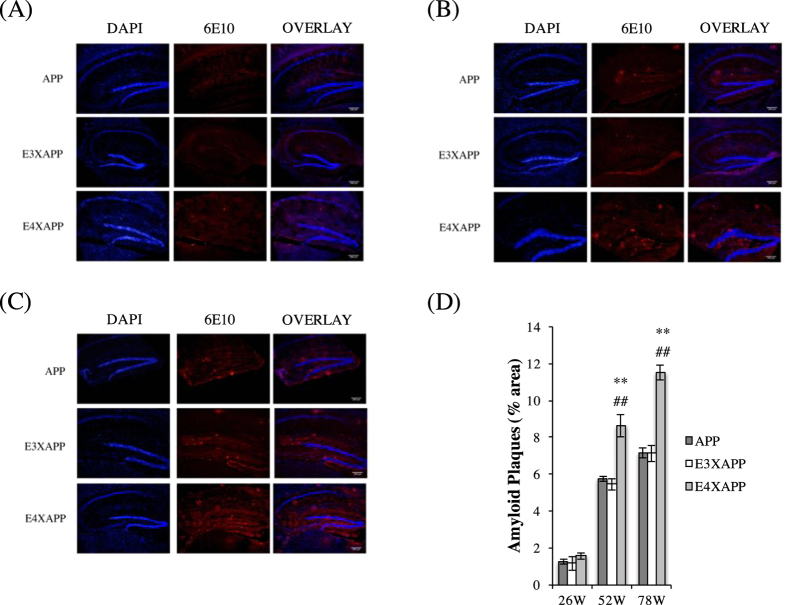
Aβ deposition in the hippocampus of aged ApoE4xAPP mice were significantly higher than APP and ApoE3xAPP mice. (**A–C**) Immunohistochemistry staining of APP, ApoE3xAPP (E3XAPP) and ApoE4xAPP (E4XAPP) hippocampus for amyloid plaques using 6E10 antibody at (**A**) 26, (**B**) 52 and (**C**)78 weeks. (**D**) Quantification of immunohistochemistry staining. At 26 weeks, no significant difference in Aβ load was detected (One-way ANOVA, F = 0.6860). At 52 and 78 weeks, higher hippocampal Aβ load was detected in ApoE4xAPP mice as compared to ApoE3xAPP and APP mice (One-way ANOVA, F = 17.64, F = 45.29, respectively). At 52 weeks, ApoE4xAPP mice have significantly more Aβ load than APP (**p < 0.01) and ApoE3xAPP (^##^p < 0.01) mice respectively (Tukey-Kramer). At 78 weeks, ApoE4xAPP mice also showed significantly more Aβ load than APP (**p < 0.01) and ApoE3xAPP (^##^p < 0.01) mice respectively (Tukey-Kramer). Each value represents the mean ± SEM for individual mouse brain sample (n = 3).

The hippocampus is the site of the brain where early Aβ deposition occurs in AD^21^, resulting in neuronal death and the impairment of cognitive faculties^1^,^22^. In light of this, we wanted to observe the extent of Aβ accumulation occurring in the hippocampus of the ageing ApoE3xAPP and ApoE4xAPP mice (Fig. 1).

At 26 weeks old, we did not detect significant differences in hippocampal Aβ load between ApoE3xAPP and ApoE4xAPP mice (One-way ANOVA, F = 0.6860) (Fig. 1A). However, as the mice aged to 52 and 78 weeks, higher hippocampal Aβ load was detected in ApoE4xAPP mice as compared to ApoE3xAPP mice (One-way ANOVA, F = 17.64, 45.29, respectively) (Fig. 1B,C). At 52 weeks, plaque load in ApoE4xAPP mice was 36% and 33% higher compared to ApoE3xAPP (^##^p < 0.01, Tukey-Kramer) and APP mice respectively (**p < 0.01, Tukey-Kramer) (Fig. 1D). At 78 weeks, plaque load in ApoE4xAPP mice was ~38% higher compared to both ApoE3xAPP and APP mice (^##^p < 0.01, **p < 0.01, respectively, Tukey-Kramer) (Fig. 1D). These results suggested that changes in Aβ burden were human ApoE isoform-dependent.

### ApoE4xAPP mice exhibited spatial memory impairment at 26 weeks

**Figure 2:**
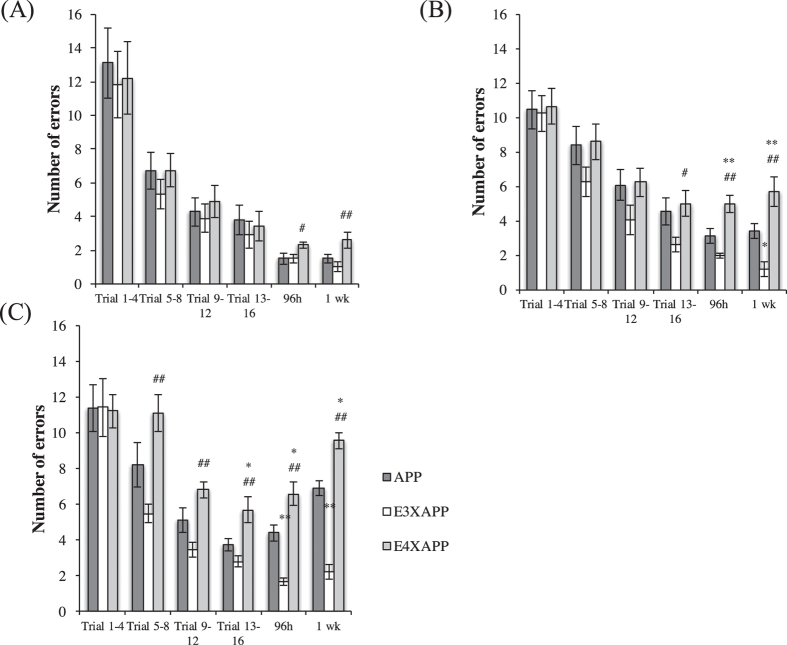
ApoE4xAPP mice exhibited spatial memory impairment at 26 weeks. (**A**) At 26 weeks, there were no significance differences in the number of errors made during the learning trials (1–16) among APP, ApoE3xAPP and ApoE4xAPP mice (One-way ANOVA, F = 0.07862, 0.5190, 0.8266, 0.2743, respectively). ApoE4xAPP mice made more errors compared to ApoE3xAPP mice during the short term (^#^p < 0.05) and long term memory trials (^##^p < 0.01) (One-way ANOVA, F = 3.368, 5.854, respectively, Tukey-Kramer). (**B**) At 52 weeks, the ApoE4xAPP mice made significantly more errors than ApoE3xAPP mice during trials 13–16 (^#^p < 0.05) (One-way ANOVA, F = 3.398, Tukey-Kramer). During the short-term probe trial, ApoE4xAPP mice made significantly more errors than APP (**p < 0.01) and ApoE3xAPP (^##^p < 0.01) mice (One-way ANOVA, F = 15.21, Tukey-Kramer) During the long-term memory probe trial, ApoE4xAPP mice made significantly more errors than APP (**p < 0.01) and ApoE3xAPP (^##^p < 0.01) mice, while ApoE3xAPP mice made significantly less errors than APP mice (*p < 0.05) (One-way ANOVA, F = 14.23, Tukey-Kramer). (**C**) At 78 weeks, ApoE4xAPP mice made more mistakes than ApoE3xAPP mice during trials 5–8 (^##^p < 0.01) and 9–12 (^##^p < 0.01) (One-way ANOVA, F = 7.127, F = 8.425, respectively, Tukey-Kramer). During trials 13–16, ApoE4xAPP mice made more errors than APP (*p < 0.05) and ApoE3xAPP mice (^##^p < 0.01) (One-way ANOVA, F = 7.834, Tukey-Kramer). During the short-term memory and long-term memory probe trials, ApoE4xAPP mice made more errors than APP (*p < 0.05) and ApoE3xAPP mice (^##^p < 0.01). However, ApoE3xAPP mice made less errors than APP mice (**p < 0.01) (One-way ANOVA, F = 26.16, F = 68.56, respectively, Tukey-Kramer). Error bars represent ± SEM (n = 7–10).

We next investigated if Aβ plaque load was correlated with spatial memory performance. Using an eight-arm Radial Maze, there were no significance differences in the number of errors made during the learning trials (1–16) among APP, ApoE3xAPP and ApoE4xAPP mice at 26 weeks old (One-way ANOVA, F = 0.07862, 0.5190, 0.8266, 0.2743, respectively). However during the short-term and long-term memory probe trials, ApoE4xAPP mice performed worse than the ApoE3xAPP mice (Fig. 2A) (One-way ANOVA, F = 3.368, 5.854, respectively. ^#^p < 0.05, ^##^p < 0.01, Tukey-Kramer).

At 52 weeks old (Fig. 2B), the ApoE4xAPP mice exhibited spatial memory deficits at the later stages (trials 13–16) of the acquisition trials and made more errors when compared to ApoE3xAPP mice (One-way ANOVA, F = 3.398, ^#^p < 0.05, Tukey-Kramer). During the short-term probe trial, ApoE4xAPP mice made significantly more errors than APP (**p < 0.01) and ApoE3xAPP (^##^p < 0.01) mice (One-way ANOVA, F = 15.21, Tukey-Kramer). During the long-term memory probe trial, ApoE4xAPP mice made significantly more errors than APP (**p < 0.01) and ApoE3xAPP (^##^p < 0.01) mice, while ApoE3xAPP mice made significantly less errors than APP mice (*p < 0.05) (One-way ANOVA, F = 14.23, Tukey-Kramer).

When the mice were at 78 weeks old, the ApoE4xAPP mice showed learning deficits after the first day of memory acquisition (Fig. 2C). ApoE4xAPP mice made more mistakes than ApoE3xAPP mice from trials 5–8 (^##^p < 0.01) and 9–12 (^##^p < 0.01) (One-way ANOVA, F = 7.127, F = 8.425, respectively, Tukey-Kramer). During trials 13–16, ApoE4xAPP mice made more errors than APP (*p < 0.05) and ApoE3xAPP mice (^##^p < 0.01) (One-way ANOVA, F = 7.834, Tukey-Kramer). During the short-term and long-term memory probe trials, ApoE4xAPP mice made more errors than APP (*p < 0.05) and ApoE3xAPP mice (^##^p < 0.01). However, ApoE3xAPP mice made fewer errors than APP mice (**p < 0.01) (One-way ANOVA, F = 26.16, F = 68.56, respectively, Tukey-Kramer).

### Insulin signaling was impaired in the ApoE4xAPP mice at 26 weeks

Insulin response in the hippocampus of AD post-mortem cases was shown to be significantly impaired^13^. However, the use of AD post-mortem tissues meant that the cellular changes that took place during disease development were not being observed. It was also unknown if insulin activity in the hippocampus was regulated by ApoE isoforms.

To observe the hippocampal response to insulin as the mice aged, we acutely treated hippocampal slices of the three mouse lines with 1 μM of insulin^15^ before probing for activated Akt expression. We chose not to probe for phosphorylated insulin receptor (IR) as an earlier study had shown that activating IR (via phosphorylation) did not translate to activated downstream insulin signaling in AD^13^. Hence, probing for downstream activation of the insulin receptor such as activated Akt was a more accurate indicator of activation of downstream insulin signaling^15^,^23^,^24^.

**Figure 3:**
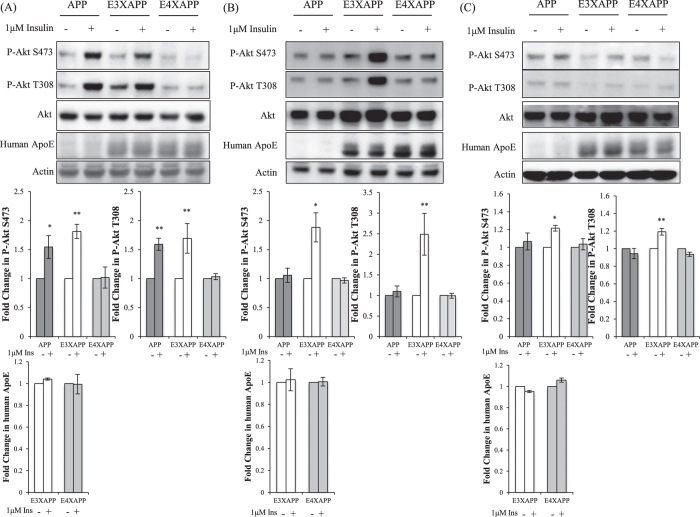
Insulin signaling was impaired in the hippocampus of 26 weeks ApoE4xAPP mice. (**A–C**) Immunoblots of hippocampal slices from APP, ApoE3xAPP (E3XAPP) and ApoE4xAPP (E4XAPP) mice were treated with or without 1 μM of insulin. Each blot is a representative of five independent experiments. Blot images were cropped for comparison and all relevant gels have been run under similar experimental conditions. Cropping area is indicated by black line surrounding the border of the blot figures. Densitometry analysis was performed using the NIH ImageJ software. Results were expressed as a fold change in protein expression as compared to the non-treated slices within the same mouse line. Error bars represent ± SEM (n = 5 slices). **(A)** At 26 weeks, the hippocampus of ApoE4xAPP mice was not sensitive to insulin stimulation. Insulin treatment resulted in a significant increase in P-Akt S473 (*p < 0.05, **p < 0.01) and P-Akt T308 (**p < 0.01) expression in ApoE3xAPP and APP mice. (**B**) At 52 weeks, both the hippocampus of ApoE4xAPP and APP mice were unresponsive to insulin stimulation. In ApoE3xAPP mice, insulin stimulation led to a marked increase in P-Akt S473 and P-Akt T308 expression (*p < 0.05, **p < 0.01). (**C**) At 78 weeks, insulin stimulation only increased P-Akt S473 and P-Akt T308 expression in ApoE3xAPP mice (*p < 0.05, **p < 0.01).

At 26 weeks, where Aβ plaque load in the hippocampus was low and comparable among APP, ApoE3xAPP and ApoE4xAPP mice (Fig. 1A), the hippocampal slices of ApoE4xAPP mice were not responsive to insulin stimulation (Fig. 3A). This coincided with the age where the ApoE4xAPP displayed spatial memory deficits (Fig. 2A). In contrast, insulin treatment resulted in >1.5-fold increase in Akt phosphorylation at S473 and T308 in ApoE3xAPP and APP mice (**p < 0.01, *p < 0.05, respectively).

At 52 and 78 weeks, both APP and ApoE4xAPP mice were unresponsive to insulin stimulation (Fig. 3B,C). However in 52 weeks ApoE3xAPP mice, insulin treatment led to a 1.8- and 2.5-fold increase in P-Akt S473 and T308 expression (Fig. 3B) (*p < 0.05, **p < 0.01, respectively). When ApoE3xAPP mice were aged to 78 weeks, insulin stimulation led to ~20% increase in P-Akt S473 and T308 expression (*p < 0.05, **p < 0.01, respectively) (Fig. 3C).

### Insulin stimulation enhances GluR1 phosphorylation by forskolin

Since spatial memory impairment was associated with insulin unresponsiveness in the hippocampus of our mouse models, we investigated if insulin stimulation could induce long-term potentiation (LTP) in the hippocampus. An increase in GluR1-containing AMPA receptors is a marker of LTP^25^,^26^,^27^,^28^,^29^,^30^, and an increase in AMPA GluR1 subunits was also positively linked to the learning capacity of the mice in the radial maze^31^.

We chemically induced LTP using 50 μM of forskolin (FSK) in the mouse hippocampal slices and probed for AMPA GluR1 phosphorylation^28^. The FSK concentration used in our experiment contains 0.1% DMSO, which had been reported to have no effect on LTP^25^,^32^.

**Figure 4:**
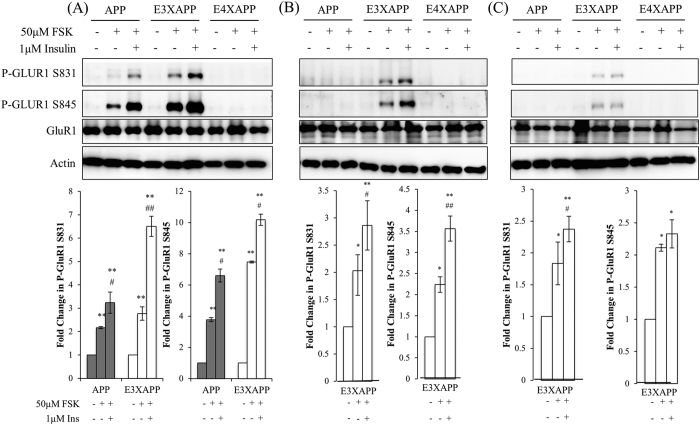
Insulin stimulation enhances GluR1 phosphorylation by forskolin. (**A–C**) Immunoblots of hippocampal slices from APP, ApoE3xAPP (E3XAPP) and ApoE4xAPP (E4XAPP) mice were treated with 50 μM of forskolin (FSK) with or without pre-treatment with 1 μM of insulin. Each blot is a representative of six independent experiments. Blot images were cropped for comparison and all relevant gels have been run under similar experimental conditions. Cropping area is indicated by black line surrounding the border of the blot figures. Results were expressed as a fold change in protein expression as compared to the non-treated slices within the same mouse line. Error bars represent ± SEM (n = 6 slices). (**A**) At 26 weeks, FSK treatment significantly increased P-GluR1 S831 (**p < 0.01) and P-GluR1 S845 (**p < 0.01) expression in ApoE3xAPP and APP mice. Insulin pre-treatment further potentiated P-GluR1 S831 and P-GluR1 S845 expression in ApoE3xAPP and APP mice when compared to non-treated slices (**p < 0.01). This insulin stimulated increase was significantly higher than FSK treatment alone (^#^p < 0.05, ^##^p < 0.01). (**B**) At 52 weeks, only slices from ApoE3xAPP mice were responsive to FSK treatment. When compared to non-treated slices within the same mouse line, FSK treatment significantly increased P-GluR1 S831 and S845 expression (*p < 0.05). Insulin pre-treatment further potentiated P-GluR1 phosphorylation in ApoE3xAPP mice (**p < 0.01) as compared to non-treated slices. When compared against slices that were treated with only FSK, insulin stimulation led to a greater increase in P-GluR1 expression in ApoE3xAPP mice (^#^p < 0.05, ^##^p < 0.01). (**C**) At 78 weeks, FSK treatment significantly increased GluR1 phosphorylation at S831 and S845 in ApoE3xAPP mice (*p < 0.05). As compared to non-treated slices, insulin pre-treatment potentiated FSK-induced GluR1 phosphorylation in ApoE3xAPP mice (*p < 0.05, **p < 0.01). When compared against slices that were treated with FSK only, insulin stimulation only led to a greater increase in phosphorylated GluR1 S831 expression in ApoE3xAPP mice (^#^p < 0.05).

At 26 weeks (Fig. 4A), hippocampal slices from ApoE4xAPP mice were not responsive to FSK treatment, and insulin stimulation did not potentiate further phosphorylation of GluR1 subunits. In contrast, FSK treatment alone led to a 2.8- and 7.5-fold increase in P-GluR1 S831 and S845 expression in ApoE3xAPP hippocampal slices (**p < 0.01). In APP hippocampal slice, FSK treatment alone led to a 2- and 3.8-fold increase in P-GluR1 S831 and S845 expression (**p < 0.01). When the ApoE3xAPP hippocampal slices were pre-treated with insulin before FSK stimulation, we observed a 6.5- and 10-fold increase in P-GluR1 S831 and S845 expression when compared to non-treated slices (**p < 0.01). Similarly, insulin pre-treatment in APP hippocampal slices led to a 3- and 7-fold increase in P-GluR1 S831 and S845 expression as compared to non-treated slices (**p < 0.01). In both APP and ApoE3xAPP mice, this insulin stimulated increase was significantly higher than FSK treatment alone (^#^p < 0.05, ^##^p < 0.01).

At 52 and 78 weeks (Fig. 4B,C), only slices from ApoE3xAPP mice were responsive to FSK stimulation. FSK treatment of 52 weeks ApoE3xAPP hippocampal slices (Fig. 4B) resulted in increased P-GluR1 S831 and P-GluR1 S845 expression as compared to non-treated slice (*p < 0.05). When the slices were pre-treated with insulin, it led to a 2.86 and 3.5-fold increase in P-GluR1 S831 and S845 expression when compared to non-treated slices (**p < 0.01). At 52 weeks, this insulin stimulated increase was also significantly higher than FSK treatment alone (^#^p < 0.05, ^##^p < 0.01).

When the ApoE3xAPP mice aged to 78 weeks (Fig. 4C), FSK treatment resulted in ~2-fold increase in P-GluR1 S831 and S845 expression in the hippocampal slices (*p < 0.05). Pre-treatment with insulin resulted in a 2.4-fold and 2.3-fold increase in P-GluR1 S831 and S845 expression, respectively, as compared to non-treated slices (*p < 0.05, **p < 0.01). However, when compared against slices that were treated with FSK only, insulin stimulation only led to a significant increase in phosphorylated GluR1 S831 expression in ApoE3xAPP mice (^#^p < 0.05). These data suggest that a functional insulin response could further potentiate LTP in the presence of Aβ.

### Insulin response increased AMPA mEPSC amplitude in ApoE3 and ApoE4 neurons

Although the majority of ApoE is secreted by glial cells, ApoE can also be produced by neurons^33^. We had previously reported significant ApoE expression in hippocampal neurons from the ApoE3 and ApoE4 mice^34^.

Using endogenous glutamate released by the presynaptic terminals of the hippocampal neurons, we examined if insulin stimulation increased LTP at cellular level by recording the AMPA mEPSCs in hippocampal neurons, and measured the changes to the amplitude and frequency of AMPA mEPSCs. MK801 was added into the external solution during recording to block out the NMDA mEPSCs. In addition, external solution containing DNQX was perfused into the recording chamber after mEPSC recording as proof-of-concept to ensure that the recorded currents were conducted by the AMPA receptor (Figure S4A,B).

When comparing among treatment groups, insulin treatment has no effect on mEPSC intervals while Aβ42 pre-treatment significantly increased mEPSC intervals in both ApoE3 and ApoE4 neurons (One-way ANOVA, F = 26.40,17.32 respectively). In both ApoE3 and ApoE4 neurons, Aβ42 (**p < 0.01) and Aβ42+Ins treated neurons (^##^p < 0.01) showed significant increases in mEPSC intervals when compared to other treatment groups (Tukey-Kramer).

**Figure 5:**
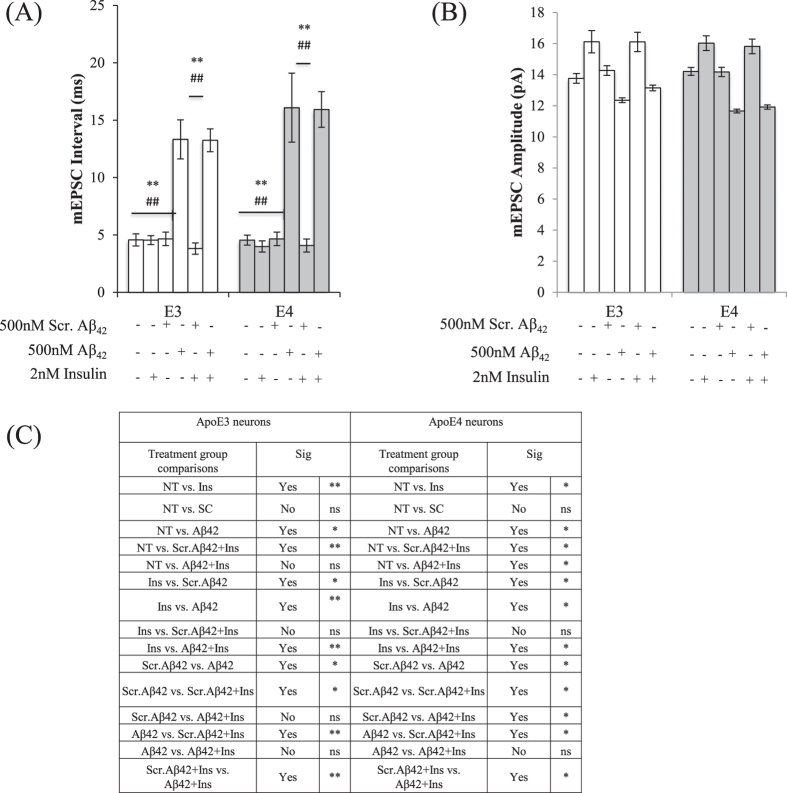
Insulin stimulation increased mEPSC amplitudes but did not rescue Aβ42-induced down-regulation of AMPA mEPSC frequencies and amplitudes in both ApoE3 and ApoE4 neurons. (**A**) mEPSC interval histograms from ApoE3 and ApoE4 hippocampal neurons. Insulin treatment has no effect on mEPSC intervals while Aβ42 pre-treatment significantly increased mEPSC intervals in both ApoE3 and ApoE4 neurons (One-way ANOVA, F = 26.40,17.32 respectively). In both ApoE3 and ApoE4 neurons, Aβ42 (**p < 0.01) and Aβ42+Ins (^##^p < 0.01) treated neurons showed significant increases in mEPSC intervals when compared to other treatment groups (Tukey-Kramer). (**B**) mEPSC amplitude histograms from ApoE3 and ApoE4 hippocampal neurons. Insulin treatment increased mEPSC amplitudes in both ApoE3 and ApoE4 neurons (**p < 0.01, *p < 0.05) (One-way ANOVA, F = 11.17, 5.722, respectively, Tukey-Kramer). (**C**) Summary of post-hoc Tukey-Kramer analysis. Error bars represent ± SEM (n = 6). NT refers to no treatment.

Adding 2 nM^13^ insulin led to a 17.8% and 12.8% increase in mEPSC amplitude in ApoE3 (**p < 0.01) and ApoE4 neurons (*p < 0.05), respectively (One-way ANOVA, F = 11.17, 5.722, respectively, Tukey-Kramer) (Fig. 5B). The presence of scrambled Aβ42 as a control did not affect this observation. In neurons pre-treated with Aβ42, the mEPSC amplitudes were reduced by 11% and 17.9% in ApoE3 (*p < 0.05) and ApoE4 (*p < 0.05) neurons respectively. When the neurons pretreated with Aβ42 were subjected to insulin stimulation, insulin could not rescue Aβ42-induced reduction in mEPSC amplitudes in both ApoE3 and ApoE4 neurons (Fig. 5C).

The Aβ42 concentration used in our study is comparable to other studies used on primary neurons^35^. The Aβ42 oligomers were predominantly trimers (Figure S2) and an age-related increase of the Aβ trimers were also detected in the brain of our ApoExAPP mice (Figure S1).

### Insulin response increased GluR1-containing AMPA receptor insertion and prevented Aβ42 downscaling of surface GluR1 subunits in ApoE3 neurons

Insulin can increase AMPA GluR1 subunit insertion^36^, and an increase in AMPA receptors is synonymous with increased synaptic strength^37^. Dynamic recycling of AMPARs in neurons can be regulated via phosphorylation^38^. As insulin stimulation increased AMPA mEPSC amplitude without increasing AMPA mEPSC frequency, we explored if the increase in mEPSC amplitude was a result of an increase in GluR1-containing AMPA receptors.

**Figure 6:**
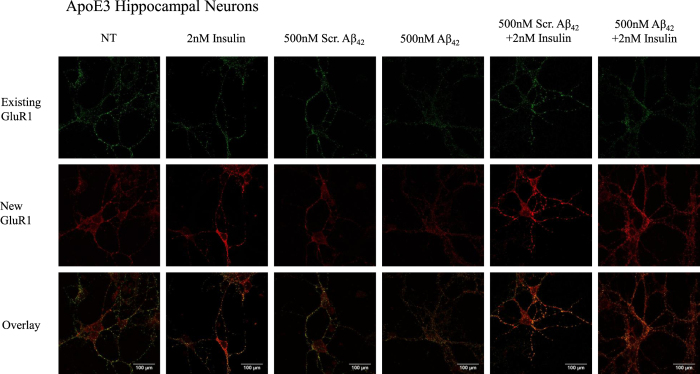
Insulin stimulation increased GluR1-containing AMPA receptor insertion and prevented Aβ42 downscaling of surface GluR1 subunits in ApoE3 neurons. Representative confocal images of immunocytochemistry staining of ApoE3 hippocampal neurons with anti-GluR1.

**Figure 7:**
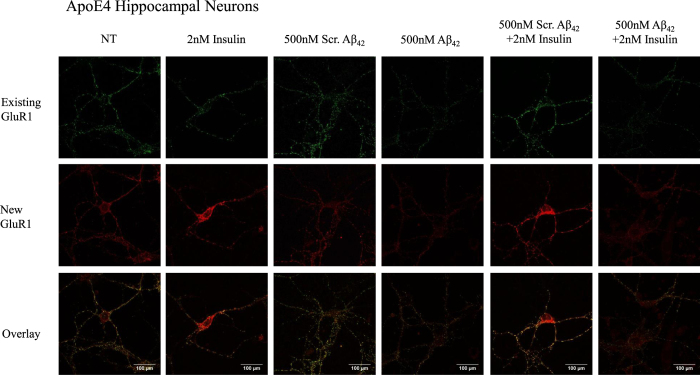
Insulin stimulation increased GluR1-containing AMPA receptor insertion in ApoE4 neurons but could not rescue Aβ42 downscaling of surface GluR1 subunits. Representative confocal images of immunocytochemistry staining of ApoE4 hippocampal neurons with anti-GluR1.

**Figure 8:**
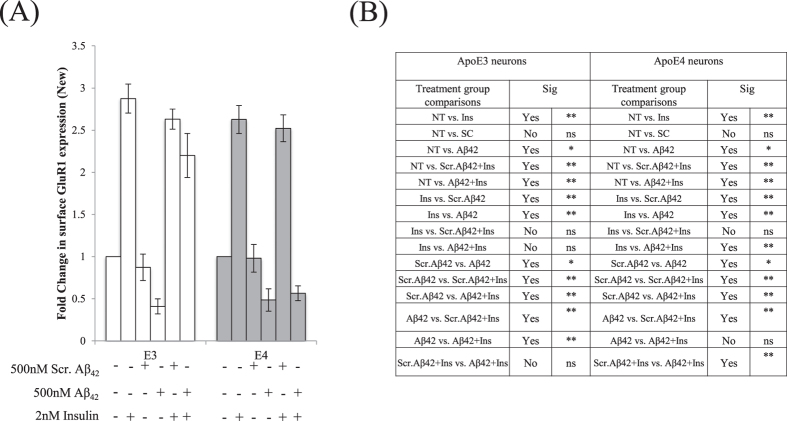
Quantification of surface GluR1-containing AMPA receptors in ApoE3 and ApoE4 neurons. Results were expressed as a fold change against non-treated neurons of the same genotype. (**A**) An increase in surface GluR1 expression by about 2.7 fold was observed following insulin treatment in ApoE3 (One-way ANOVA, F = 19.18) and ApoE4 neurons (One-way ANOVA, F = 13.48). Pre-treatment of neurons with Aβ42 reduced new GluR1 expression in ApoE3 and ApoE4 neurons by about 2-fold (*p < 0.05). Insulin stimulation increased surface GluR1-containing AMPA receptors in ApoE3 neurons pre-treated with Aβ42 by about 2.3 fold (**p < 0.01). (**B**) Summary of post-hoc Tukey-Kramer analysis. Error bars represent ± SEM (n = 6).

We differentially stained the ApoE3 (Fig. 6) and ApoE4 (Fig. 7) neuron surface for existing and newly inserted AMPA GluR1 subunits^39^. An increase in surface GluR1 expression by about 2.7 fold was observed following insulin treatment in ApoE3 (One-way ANOVA, F = 19.18, **p < 0.01, Tukey-Kramer) (Figs 6 and 8) and ApoE4 neurons (One-way ANOVA, F = 13.48 **p < 0.01) (Figs 7 and 8). As compared to non-treated (NT) neurons, pre-treatment of neurons with Aβ42 reduced new GluR1 expression in ApoE3 and ApoE4 neurons by about 2-fold (*p < 0.05) (Figs 6, 7, 8). Insulin stimulation increased surface GluR1-containing AMPA receptors in ApoE3 neurons pre-treated with Aβ42 by about 2.3 fold (**p < 0.01) (Figs 6 and 8). However, in Aβ42 pre-treated ApoE4 neurons, insulin stimulation could not rescue Aβ42 reduction in surface GluR1-containing AMPA receptor expression (Figs 7 and 8).

## Discussion

Insulin could affect cognition in an ApoE isoform dependent manner^14^,^17^, and insulin impairment was detected in postmortem AD brain samples^12^,^13^. While it was known that high Aβ levels could lead to insulin signaling impairment^15^, those experiments were not done in the presence of human ApoE. Hence, it was unclear if ApoE isoforms could have modulated insulin sensitivity and memory decline in AD. Here, we showed that Aβ-mediated memory decline in the hippocampus was associated with insulin sensitivity, which in turn was regulated by the ApoE isoform expressed.

Our data showed that insulin signaling was impaired in 26 weeks ApoE4xAPP hippocampus at comparable plaque load to the other 2 strains. This insulin signaling impairment was highly associated with spatial memory deficits in the ApoE4xAPP mouse, and these defects could have originated from earlier abnormalities in the posteromedial cortex (PMC)^10^ and/or entorhinal cortex^40^, as seen in ApoE4 subjects. Insulin signaling impairment in ApoE4xAPP mice also prevented further enhancement of phosphorylated GluR1-containing AMPA receptors, where the phosphorylation of GluR1 residues S831 and S845 increased membrane trafficking, and receptor opening and conductance^25^,^28^,^30^,^38^. Unlike the ApoE4xAPP mouse, the ApoE3xAPP hippocampal slices remained responsive to insulin as the mice aged. This was associated with better spatial memory performance and enhanced phosphorylation of GluR1-containing receptors. While the protection of memory by insulin seemed to decrease with age in ApoE3xAPP mice, it was expected given the age-related increase in plaque load.

A recent study had associated the impaired memory performance of ApoE4 expressing APP mice models to reduced synaptic protein expression^8^. However, receptor function is robust and dynamic, and simply observing basal protein expression from brain lysates cannot capture the activated response of these receptors to stimulation.

We had previously reported that ApoE4 expression alone could not impair insulin signaling but the co-presence of ApoE4 and Aβ42 could attenuate downstream insulin response in hippocampal neurons^34^. Here, we further show that insulin response increased AMPA mEPSC amplitude in both ApoE3 and ApoE4 neurons. While insulin stimulation did not increase AMPA mEPSC amplitude in Aβ42 pre-treated neurons, this could be a reflection of reduced glutamate availability from the presynaptic terminal, as insulin stimulation could not rescue Aβ42-mediated attenuation of mEPSC frequencies.

A previous study had shown that insulin resulted in GluR1 removal from the cell surface^41^. However, the insulin concentration applied to the neuronal culture was not physiological^13^. At near physiological concentrations in cell cultures, insulin was shown to increase GluR1 insertion^27^.

Our results showed that this insulin-mediated GluR1 insertion was ApoE isoform-dependent. While insulin stimulation and Aβ42 pre-treatment led to comparable increases and reductions in GluR1 subunits in both ApoE3 and ApoE4 neurons, insulin stimulation could not increase new GluR1 subunit expression in ApoE4 neurons pre-treated with Aβ42. On the other hand, insulin stimulation led to insertion of GluR1-containing AMPA receptors in Aβ42 pre-treated ApoE3 neurons, suggesting that insulin action is postsynaptic and promoted GluR1-containing AMPA receptor insertion in an ApoE isoform dependent manner.

Knocking out ApoE in mutant APP mice can significantly reduce amyloid deposition^42^,^43^,^44^. Hence, introducing human ApoE to mouse ApoE knockout-APP mice will preclude timely analyses of ApoE isoform specific on the onset and progression Aβ pathology^45^,^46^. This major drawback can be addressed by crossing with APP mice (5xFAD) with rapid-onset of Aβ pathology^47^. While most mutant APP mice develop plaques around 6–8 months, the 5xFAD mice develop plaques by 2 months^9^.

Unlike other studies^45^,^46^,^48^, we decided to keep the endogenous mouse ApoE while introducing the human ApoE in the ApoE3xAPP and ApoE4xAPP mice. This approach did not delay Aβ deposition in our ApoExAPP mice^34^.

Our results also show that amyloid deposition and memory decline in the control APP mice are intermediate between the ApoE3xAPP mice and ApoE4xAPP mice. This is unlikely due to the endogenous mouse ApoE since the APP mice express twice the amount of mouse ApoE as compared to ApoE3xAPP and ApoE4xAPP (Figure S3).

Similar to other studies^47^,^48^, our ApoE4 expressing APP mice have higher amyloid deposition than ApoE3xAPP mice^34^. The changes in hippocampal Aβ content shown in this study may due to reduced ApoE4 expression in the aged mice^34^,^49^,^50^ since increasing ApoE4 expression can reduce amyloid pathology^51^,^52^.

However, this study has several limitations. Firstly, we have only examined ApoE3 and ApoE4 but not ApoE2. Although ApoE2 is uncommon^4^,^53^, this allele is reported to have a protective effect. Hence, it will be interesting to investigate and compare how ApoE2 regulates the role of Aβ on insulin-stimulated AMPA receptor function. Secondly, we have only examined female mice. This is because female ApoE4 mice display greater cognitive impairment than male ApoE4 mice^54^,^55^,^56^. Moreover, mutant APP female mice bear heavier amyloid burden and display greater behavioral deficits^57^,^58^. In humans, ApoE4 was also reported to confer greater risk in women developing AD^59^. Therefore, it was important to determine the gender effect of ApoE on brain insulin signaling and AMPA receptor function.

Taken together, we propose an isoform-specific ApoE effect on insulin response in the presence of Aβ42 as a mechanism underlying earlier cognitive impairment observed in ApoE4 subjects. This regulation enabled ApoE3 but not ApoE4 expression, to attenuate Aβ42 inhibition of AMPA receptor function and to delay memory decline. Our work underscores the importance of connecting ApoE genotype and insulin sensitivity as an early marker for AD-related cognitive decline.

## Materials and Methods

### Animals

All experiments involving the use of live animals were conducted in accordance with the guidelines of the Institutional Animal Care and Use Committees (IACUC) and the protocols R13-4468 and BR13-4458 were approved by IACUC at the National University of Singapore.

The generation of the ApoE3xAPP and ApoE4xAPP mice were described^34^ by crossing the human ApoE3 and ApoE4 targeted replacement mice^18^,^19^ with the APP J20 transgenic mice^20^. The APP J20 mice carry a mutant human APP gene bearing the Swedish (K670N/M671L) and Indiana (V717F) mutations. All mouse lines are on C57/B6 background.

Both ApoE3xAPP and ApoE4xAPP mice are heterozygotes for the human ApoE and mouse ApoE3. The APP mice express twice the amount of mouse ApoE as compared to ApoE3xAPP and ApoE4xAPP (Figure S3). In this study, the APP J20 mice were used as controls to examine the background influence of mouse ApoE.

All mice in the study were housed conventionally, under ambient conditions (12 hrs dark, 12 hrs light). They were kept on 2018 Teklad Global 18% Protein Rodent Diet (Harland Laboratories). All experiments were performed on female APP, ApoE3xAPP and ApoE4xAPP mice at 26, 52 and 78 weeks of age.

### Radial Maze

The mice used in the Radial Maze^60^ were followed longitudinally from 26 to 78 weeks. Mice were first put through two days of habituation trial, where they were allowed to roam around the maze for 10 minutes. Novel food (Kellogg’s Fruit Loops) were scattered throughout the maze on the first day. On the second day of habituation, the novel food was restricted to the concave food bowls. Before each day of the acquisition trials, mice were fasted the night before, with water *ad libitum*.

After habituation, mice were put through a total of 16 acquisition trials, which took place over a span of four days. During the acquisition trials, 4 arms were baited with food (a small piece of a fruit loop, and placed in the concave food plate), and the other 4 arms were not baited. Throughout the entire project, the baited and non-baited arms remained the same. The mice were given 5 minutes for each trial to locate the food. After each trial, the maze was cleaned thoroughly with ethanol, uneaten food was left intact and eaten food was not replaced.

After the 16^th^ trial, the mice were rested for 96 hours and subjected to the short-term memory probe trial. The mice were given only one attempt during the short-term memory probe trial for 5 minutes. After the short-term memory probe trial, mice were rested for one week before being put through the long-term memory probe trial. The long-term memory probe-trial was similar to the short-term memory probe trial, where mice were only given one attempt for 5 minutes. During all the trials, the number of entries into a non-baited arm was noted as a memory error. The mice were considered to have entered an arm if all four limbs were inside the arm.

### Antibodies

The primary antibodies used in this study were anti-human ApoE (Calbiochem, Cat#178479), anti-human ApoE (Santa Cruz, Cat#SC98573), anti-mouse ApoE (Santa Cruz, Cat#SC6384) anti-P-Akt (S473) (Cell Signal Tech, Cat#4060), anti-P-Akt (T308) (Cell Signal Tech, Cat#2965), anti-Akt (Cell Signal Tech, Cat#4691), anti-Actin (Sigma, Cat#A2066), anti-Aβ (1–17) (6E10) (Covance, Cat#SIG-39300), anti-GluR1 (Cell Signal Tech, Cat#8850), anti-P-GluR1 (S845) (Cell Signal Tech, Cat#8084) and anti-P-GluR1 (S831) (Santa Cruz Biotech, Cat#SC-16313-R).

### Immunohistochemistry (IHC)

Sagittal sections of perfused brains were first treated with 5 M guanidine hydrochloride, pH 8, for 30 minutes and permeabilized with 0.1% Triton X for 30 minutes. The sections were blocked with 10% fetal bovine serum (FBS) (Life Technologies) for one hour and incubated with primary antibody for overnight at 4 °C. The sections were then washed and stained with secondary antibody for one hour at room temperature and the nuclei were stained with DAPI. The sections were visualized using an Olympus fluorescence microscope and images were captured at 10X.

### Western Blot and Densitometry Analysis

Mouse brain homogenates were prepared as described in our earlier study^7^, with ice-cold 1x RIPA lysis buffer (Cell Signaling Technology) containing detergents (1% Nonidet P40 and 1% sodium deoxycholate) with the protease inhibitor cocktail tablet (Roche). Tissue lysates were collected for protein quantification using BCA analysis (ThermoFischer).

The brain lysates were run using a 10% Tris-glycine polyacrylamide gel or NuPAGE^®^ Bis-Tris gels (Life Technologies), and transferred onto nitrocellulose membrane before probing with specific primary and secondary antibodies. The protein bands were visualized by chemiluminescence on the Image Station 4000R (Carestream Health Inc).

Densitometry analysis of the bands was performed by measuring the optical densities of the targeted protein bands relative to the β-actin level from the same brain sample. For protein phosphorylation, the optical densities of the phosphorylated protein bands were measured relative to the targeted total protein level from the same brain sample. The analysis was performed using the NIH Image J software.

### Hippocampal Slice preparation and treatment

Hippocampus from the right hemisphere was isolated and 400 μm thick transverse slices were prepared as described elsewhere^61^. The slices were allowed to recover for 2 hours in artificial cerebrospinal fluid (aCSF) bath maintained at 32 °C and continuously bubbled with 95% oxygen and 5% carbon dioxide (Carbogen). The aCSF comprised of a modified Krebs-Ringer solution containing: 124 mM NaCl, 4.9 mM KCl, 1.2 mM KH_2_PO_4_, 2 mM MgSO_4._7 H_2_O, 2 mM CaCl_2._2 H_2_O, 24.6 mM NaHCO_3_, and 10 mM D-glucose. The pH of aCSF was between 7.3–7.4 when bubbled with carbogen^32^. For insulin treated samples, after the recovery period the slices were acutely treated with 1 μM of insulin (Sigma Aldrich, Cat#I9278) for 30 minutes and collected. In forskolin (FSK) treated experiments, FSK (Tocris, Cat#1099) was dissolved in DMSO and subsequently diluted to 50 μM in the aCSF. Final concentration of DMSO in the aCSF was kept at 0.1%, which had been reported to not affect the basal synaptic responses^62^. The slices were incubated with FSK for 20 minutes. After the respective treatments, the slices were immediately snap frozen in liquid nitrogen and stored at −80 °C for further use.

### Aβ42 preparation

Lyophilized Aβ42 and scrambled Aβ42 (1^st^ Base) were purchased and stored at −80 °C. Aβ42 and scrambled Aβ42 used in the experiments were dissolved in DMSO to yield a concentration of 500 μM, and immediately diluted to 500 nM in culture medium for use in the hippocampal neurons.

### Primary Hippocampal Neuron Culture

Hippocampal neurons were obtained from dissection of P0 pups of either ApoE3 or ApoE4 mice and digested as previously described^63^ using the Papain Dissociation System (Worthington Cat# LK003150). These hApoE mice are created on mouse ApoE knock-out (KO) background^18^,^19^ and do not express mouse ApoE. The hippocampus was dissected and titurated 10 times in papain solution and incubated at 37 °C for 40 minutes. The hippocampi were then spun at 1000 rpm, 4 °C for 10 minutes. After removing the supernatant, the pellet was added to the STOP solution (DNase, papain inhibitor and Earles’s Balanced Salt Solution, EBSS). The hippocampi were then titurated and left at room temperature for 10 minutes. The mixture was layered on top of the 10/10 solution (Bovine Serum Albumin, BSA and trypsin inhibitor in EBSS) and then spun at 1000 rpm, 4 °C for 10 minutes. The hippocampal cultures were plated in culture plate wells previously coated with poly-L-lysine (Sigma Aldrich), and maintained in Neurobasal media (Life Technologies) supplemented with B27, L-glutamine and Penicillin-Streptomycin (Life Technologies). Ara-C (Sigma Aldrich) was added to the culture after 3 days *in vitro* (DIV).

### Insulin starvation of hippocampal neurons

ApoE3 or ApoE4 hippocampal neurons were incubated with either 500 nM of Aβ42 or scrambled Aβ42 24 h prior to starvation. The Aβ42 concentration used in this study is comparable to other studies using primary neurons^35^. On the day of the experiment, the Neurobasal media (Life Technologies) was removed from the wells and the neurons were starved in Earle’s Balanced Salt Solution (EBSS) (Sigma Aldrich) for 2 hours.

### Electrophysiology

Whole-cell patch clamp recordings were done on DIV11-12 hippocampal neurons. Each neuron was recorded for at least 20 minutes with no electronic compensation. The neurons were continuously perfused with extracellular mEPSC solution using a multi-barrel perfusion system. The extracellular mEPSC solution contained 140 mM NaCl, 4 mM CaCl_2_, 5 mM KCl, 25 mM HEPES, 0.01 mM Glycine, 0.0005 mM TTX, 0.001 mM Strychnine, 0.02 mM bicuculline methiodide, 0.005 mM MK801, pH 7.2–7.5, and osmolarity of 320–330 mosmol^−1 ^64. The intracellular solution contained 140 mM CsCl_2,_ 2.5 mM EGTA, 2 mM MgCl_2_, 10 mM HEPES, 2 mM TEA, 4 mM Mg_2_ATP, pH 7.2–7.4, and osmolarity of 290–300 mosmol^−1^. MK801 was added into the mEPSC solution just before perfusion into the recording chamber. For insulin stimulated recordings, neurons were serum starved for 2 hours in EBSS and acutely treated with insulin for 30 minutes in mEPSC solution without MK801. Insulin was also added to the mEPSC solution in another barrel and perfused into the recording chamber, and maintained throughout recording. DNQX (0.005 mM) was also added into the mEPSC solution in another barrel and applied when required.

### Surface staining of AMPA receptors

DIV11-12 hippocampal neurons plated on coverslips were stained for surface AMPA receptors^39^. Neurons were incubated with either 500 nM of Aβ42 or scrambled Aβ42 24 hours prior to starvation. On the day of experiment, existing GluR1 receptors were first labeled using anti-GluR1. After one hour, the antibody was washed away and labeled with Alexa Fluor 488 (Life Technologies). The neurons were then serum starved in EBSS for 2 hours and acutely treated with insulin for 30 minutes in mEPSC solution without MK801. The cells were washed and fixed with 4% Formaldehyde and blocked with 10% FCS. Excess, unlabeled antibody was blocked with anti-Rabbit IgG. The neurons were labeled with anti-GluR1 under non-permeabilizing conditions and labeled with Alexa Fluor 568 (Life Technologies). Coverslips were mounted onto a microscope slide and fluorescent images were captured using a Zeiss LSM-510 laser-scanning confocal microscope at 63X.

### Statistical analysis

Statistical significance was calculated using two-tailed Student’s T-test or One-way ANOVA with post-hoc Tukey-Kramer analysis (Chan *et al.*^34^).

## Additional Information

**How to cite this article**: Chan, E. S. *et al.* ApoE4 expression accelerates hippocampus-dependent cognitive deficits by enhancing Aβ impairment of insulin signaling in an Alzheimer’s disease mouse model. *Sci. Rep.* **6**, 26119; doi: 10.1038/srep26119 (2016).

## Supplementary Material

Supplementary Information
