# Supplementary material for: ApoE4 expression accelerates hippocampus-dependent cognitive deficits by enhancing Aβ impairment of insulin signaling in an Alzheimer’s disease mouse model
Source: Sci Rep. 2016 May 18;6:26119. doi: 10.1038/srep26119 (PMC4870502; doi:10.1038/srep26119)
Supplement: Supplementary Information [file srep26119-s1.pdf]

Supplementary Information

ApoE4 expression accelerates hippocampus-dependent cognitive deficits by enhancing Aβ impairment of insulin signaling in an Alzheimer's disease mouse model.

Running Title: ApoE4 accelerates Aβ-linked insulin dysfunction and memory decline.

Elizabeth S Chan[1], Mahesh Shivarama Shetty[1,3], Sreedharan Sajikumar[1,3], Christopher Chen[2,4], Tuck Wah Soong[1,3] and Boon-Seng Wong [1, +]

Departments of Physiology [1] and Pharmacology [2], Yong Loo Lin School of Medicine, National University of Singapore, Singapore 117597.

[3] Memory Networks Program, Neurobiology and Ageing Program, Life Sciences Institute, National University of Singapore, Singapore 117456.

[4] Memory Ageing and Cognition Centre (MACC), National University Health System (NUHS), Singapore 117599.

[+] Corresponding author:

Dr. Boon-Seng WONG. Department of Physiology. National University of Singapore. 2 Medical Drive MD9. Singapore 117597. Singapore. Email: bwong@nus.edu.sg.

Figure S1. APP and Aβ expression in the ageing ApoExAPP mice. Western blot analysis of amyloid load in whole brain lysate of APP, ApoE3xAPP and ApoE4xAPP mice. The immunoblot was a representative of four experiments using different mouse brain samples (n=4). Blot images were cropped for comparison and all gels were run under similar conditions.

Figure S2. Aβ42 oligomer peptides added to primary hippocampal neurons. Western blot of the prepared Aβ42 peptide solutions used in hippocampal neurons.

Figure S3. Mouse ApoE expression in the ageing ApoExAPP mice. (A) Western blot analysis of mouse ApoE (mApoE) expression in the brain of APP, ApoE3xAPP (E3XAPP) and ApoE4xAPP (E4XAPP) mice at 78 weeks old. β-actin was immunoblotted to ensure similar gel loading of the starting material in each sample. The immunoblot was a representative of four experiments using different mouse brain samples (n=4). Blot images were cropped for comparison and all relevant gels have been run under similar experimental conditions. Cropping area is indicated by black line surrounding the border of the blot figures. (B) Real-time PCR analysis of mouse ApoE expression in the brain of APP, ApoE3xAPP (E3XAPP) and ApoE4xAPP (E4XAPP) mice at 78 weeks old. Mouse ApoE was significantly lowered in both ApoE3xAPP and ApoE4xAPP as compared to APP (* $p<0.05$). No significant different was detected between ApoE3xAPP and ApoE4xAPP. Results were expressed as a fold change in mRNA expression as compared to the APP mouse brain samples. Error bars represent ±SEM (n=3).

Figure S4. mEPSC traces of ApoE3 and ApoE4 neurons. (A) mEPSC traces of ApoE3 and (B) ApoE4 neurons with and without DNQX. DNQX was added to ensure the recorded currents were AMPA mEPSCs.

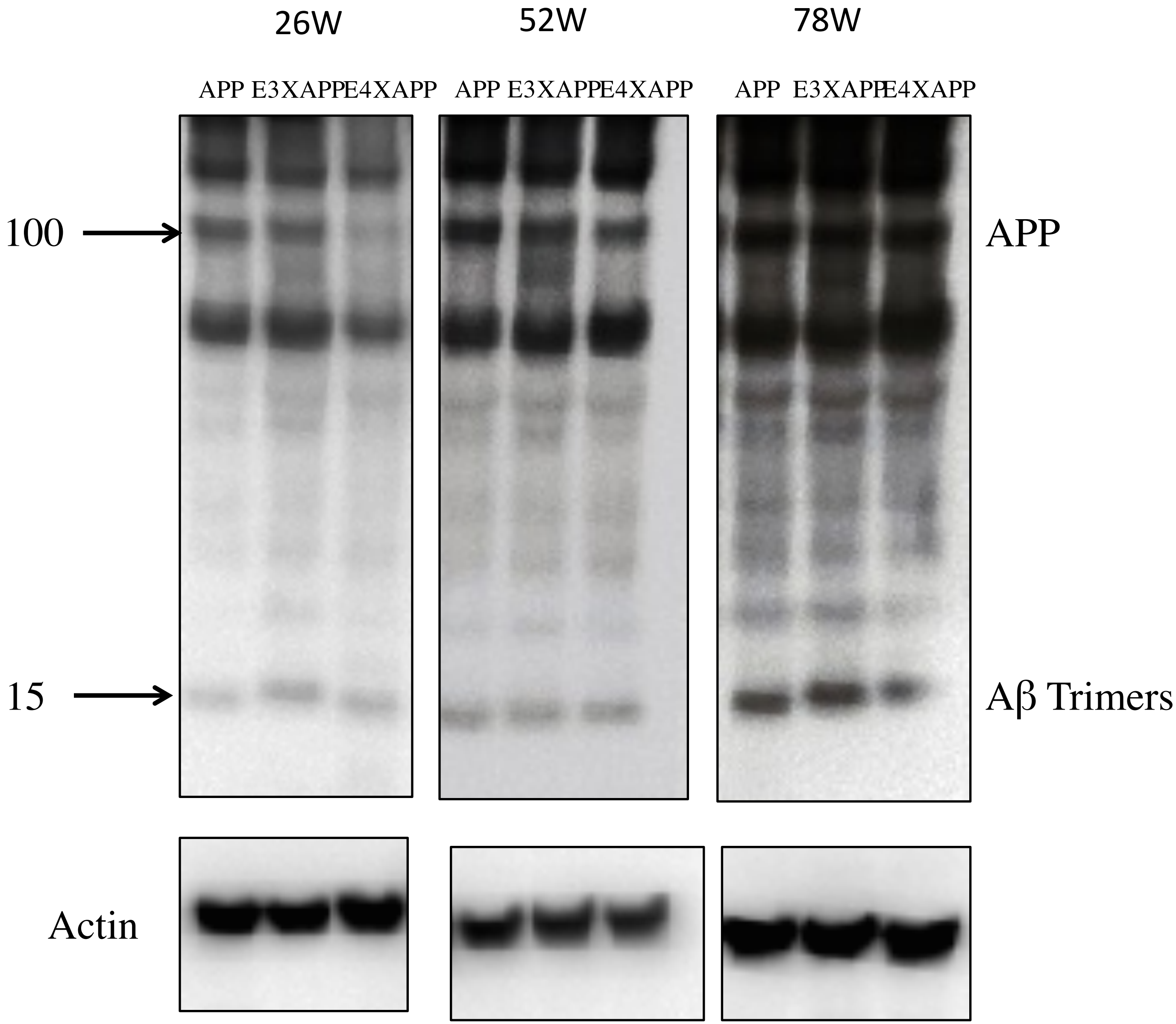

Fig S1 (Chan et al)

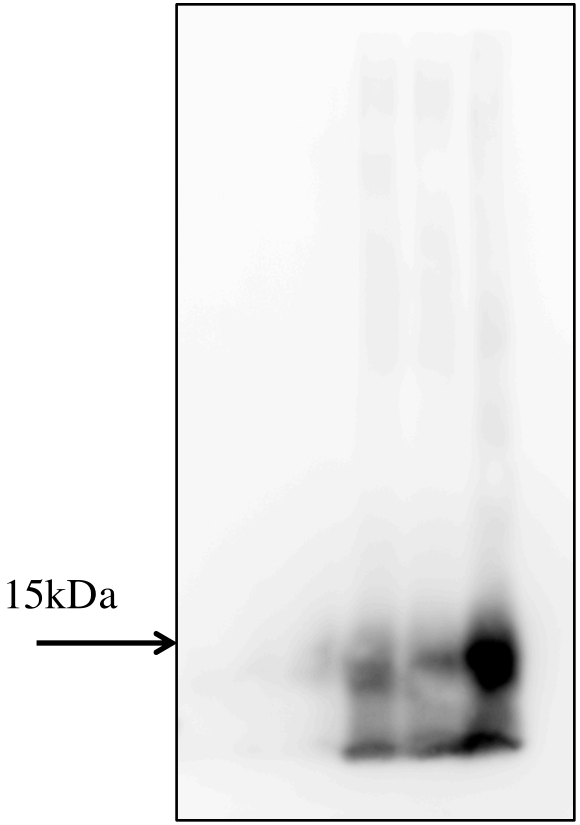

Different volume of Aβ42 peptide solutions

Fig S2 (Chan et al)

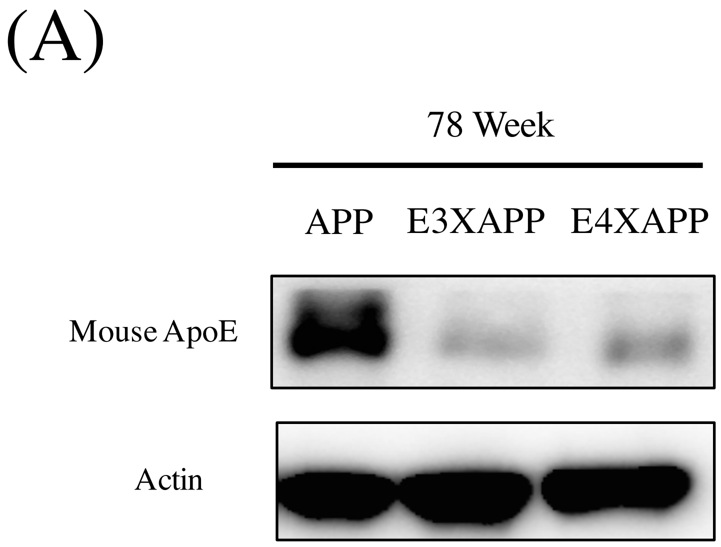

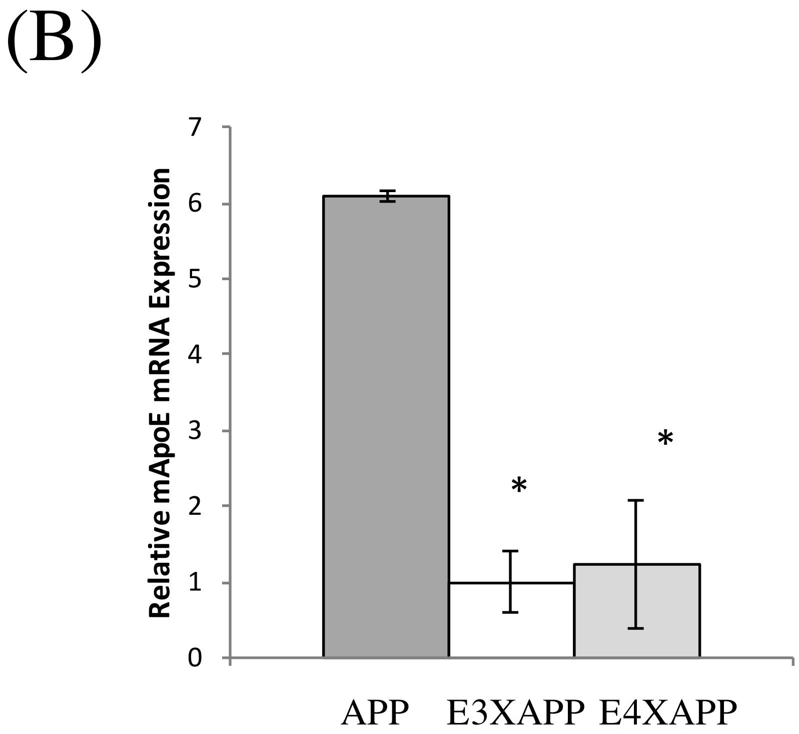

Fig S3 (Chan et al)

(A)

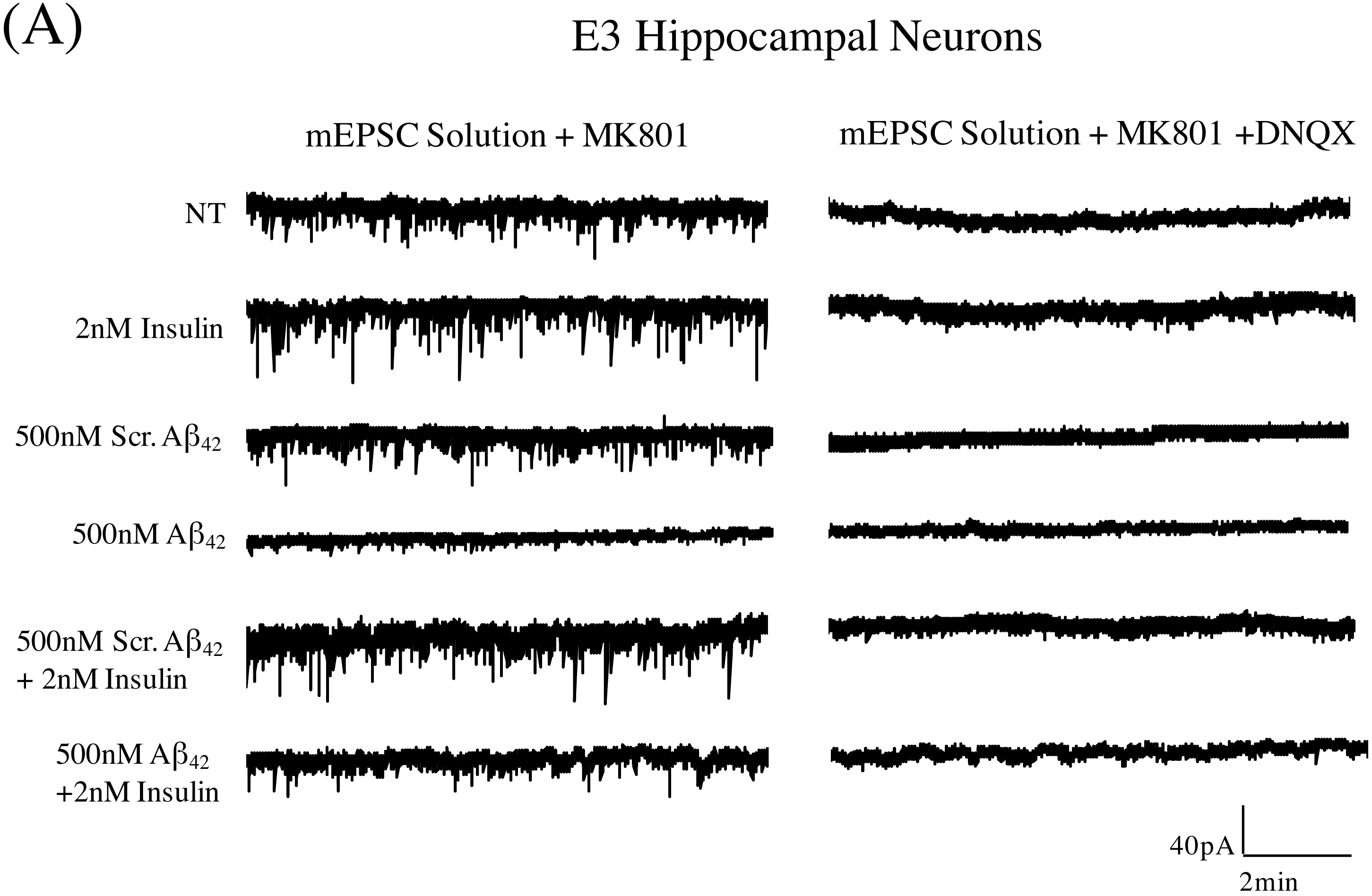

Fig S4A (Chan et al)

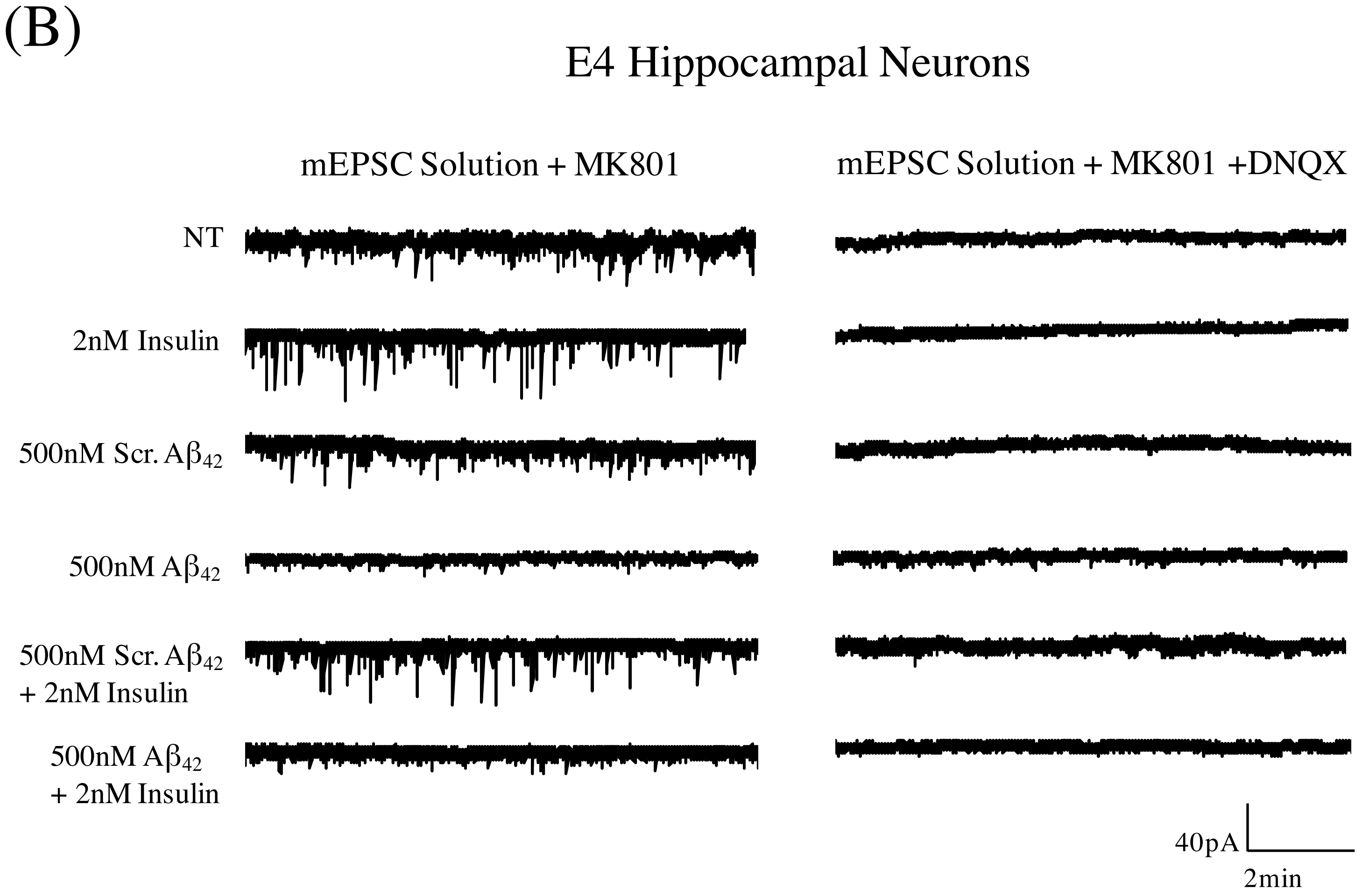

Fig S4B (Chan et al)
